# Prognostic relevance of autophagy-related markers p62, LC3, and Beclin1 in ovarian cancer

**DOI:** 10.3325/cmj.2022.63.453

**Published:** 2022-10

**Authors:** Ljubiša Jovanović, Aleksandra Nikolić, Sandra Dragičević, Milena Jović, Radmila Janković

**Affiliations:** 1 Department of Pathology and Medical Cytology, University Clinical Center of Serbia, Belgrade, Serbia; 2 Laboratory for Molecular Biology, Institute of Molecular Genetics and Genetic Engineering, University of Belgrade, Belgrade, Serbia; 3 Institute of Pathology and Forensic Medicine, Military Medical Academy, Belgrade, Serbia; 4 Institute of Pathology, Faculty of Medicine, University of Belgrade, Belgrade, Serbia

## Abstract

**Aim:**

To analyze the expression of autophagy markers p62, LC3, and Beclin1 in ovarian cancer tissue and evaluate the prognostic potential of these markers.

**Methods:**

The study enrolled 328 patients: 122 with epithelial ovarian carcinoma, 42 with atypical proliferative tumor, and 164 with benign epithelial ovarian tumor. The expression of p62, LC3, and Beclin1 was analyzed in central and invasive tumor segments with immunohistochemistry combined with tissue microarray. The expression levels of the analyzed markers were correlated with relevant histopathology parameters.

**Results:**

The expression of all analyzed markers was most remarkable in epithelial ovarian carcinoma. There was a strong positive correlation between the expressions of p62 and LC3, while these two markers negatively correlated with Beclin1. High-grade serous carcinoma had higher p62 and LC3 levels, and lower Beclin1 levels than other tumor types. This expression profile was also observed in more advanced tumor stages.

**Conclusion:**

Prominent p62 and LC3 expression in combination with weak Beclin1 expression in high-grade serous carcinoma indicates potential for the application of autophagy inhibitors in patients with this tumor subtype.

Epithelial ovarian cancer (EOC) is the fifth most common lethal gynecologic cancer, with the five-year relative survival rate of 48% (1). This highly aggressive neoplasm is frequently resistant to current therapy protocols based on various chemotherapeutic drugs. About 25% of women with ovarian cancer have innate platinum-refractory disease (2). Most patients experience disease recurrence and undergo several lines of treatment. Regardless of therapy, approximately 80% of women with advanced stages of ovarian cancer have poor overall survival (3). Ovarian cancer research is focused primarily on high-grade serous ovarian cancer (HGSC), the most common ovarian cancer histology type, accounting for 70% of all ovarian cancer cases. Patients with this highly aggressive form of ovarian cancer often show resistance to therapy and have an overall poor prognosis (2,4). Other ovarian tumors of epithelial origin include atypical proliferative tumors (APT) and benign ovarian tumors (BOT). APT, as low-grade malignant ovarian neoplasms, lead to a much better clinical outcome than EOC. These noninvasive tumors of uncertain malignant potential with specific histology pattern are considered an intermediate stage between BOT and EOC.

Among chemoresistance mechanisms in ovarian cancer, autophagy stands out as the most promising target to overcome resistance. Autophagy is a basic cellular mechanism that coordinates various physiological processes, such as cell differentiation, cell starvation, and cell survival under stress conditions, through the regulation of numerous transcription factors and signaling pathways (5). Autophagy is highly significant for the initiation and progression of neoplastic processes, as well as for tumor response to therapy (6,7). In early tumorigenesis, autophagy exerts a tumor-suppressing function by promoting genomic stability and inhibiting inflammation. During the later stages of tumor development, cancer cells use autophagy to survive the lack of nutrients and oxygen, especially in the central parts of the tumor, which are usually less vascularized (6,8,9). The ability of ovarian cancer cells to activate the autophagy mechanism and make the tumor more aggressive significantly contributes to a poor therapy response. Therefore, the use of autophagy inhibitors is a promising strategy to achieve a better outcome in these patients (6,10,11).

The most commonly studied autophagy markers are p62, LC3, and Beclin1. The expression levels of these proteins reflect the activity of the autophagic mechanism in the tumor (5). p62 protein is primarily defined as a mediator of the NF-κβ signaling pathway involved in various crucial intracellular processes, such as the regulation of oxidative stress and cellular metabolism (12). LC3 protein, with its dominant subunit B, is a component of autophagosomal membrane, and its higher expression levels may reflect an increased number of autophagosomes (13). Both p62 and LC3 are degraded during the autophagy process, and their expression may reflect the autophagy status of the tumor cells (5,13,14). Beclin1 has a tumor suppressor function in ovarian cancer, and its loss occurs in 50% of cases. The interaction of Beclin1 with VPS34 is critical for autophagy regulation (5,15).

Autophagy is under-studied in ovarian tumors. Therefore, the aim of the study was to assess the expression of autophagy markers p62, LC3, and Beclin1 in ovarian cancer tissue and evaluate their prognostic potential.

## Material and methods

### Study cohort

The study enrolled patients who underwent surgical removal of epithelial ovarian tumors at the Clinic for Gynecology and Obstetrics, University Clinical Center of Serbia, Belgrade, in the period 2017-2019. Of 328 patients, 122 had epithelial ovarian carcinomas (EOC), 42 had atypical proliferative tumors (APT), and 164 had benign ovarian tumors (BOT). The pathological classification of tumor stage was performed according to the International Federation of Gynecology and Obstetrics. We gathered data on age, histological type of tumor, tumor differentiation, stage, menopausal status, presence of lymphovascular tumor invasion, necrosis, and intratumoral and peritumoral lymphocyte infiltration. Exclusion criteria were secondary ovarian tumors, ovarian tumors from non-epithelial origin, and age younger than 18 years. The study was approved by the Ethics Committee of the University Clinical Center of Serbia. All participants gave informed consent.

### Tissue microarray

The tissue microarray (TMA) was constructed by using a 3-mm puncture needle from formalin-fixed and paraffin-embedded tumor samples. The central part of EOC represented the first cylinder, while the peripheral, invasive tumor part represented the second cylinder. From APT and BOT, we chose one cylinder per sample. The recipient paraffin block was constructed as a set of 28 cylinders (16). Covering trophoblast and syncytiotrophoblast of the placental tissue served as a positive control for immunostaining (17). In the first row of each block, placental tissue was placed for block orientation.

### Immunohistochemical analysis

Immunohistochemical analysis of p62, LC3, and Beclin1 markers was performed on TMA sections with Autostainer Link 48 (Agilent, Glostrup, Denmark). EnVision FLEX epitope unmasking solution, pH 6.1 (K8005, Agilent), was used for LC3B antibody epitope unmasking. EnVision FLEX epitope unmasking solution, pH 9.0 (K8004, Agilent), was used for p62 and Beclin1 antibody epitope unmasking. Immunohistochemical analysis was performed with the visualization system EnVision FLEX (Agilent). The following primary antibodies were used: polyclonal rabbit anti-human p62 antibody (ab155686, Abcam, Cambridge, UK) at 1:500 dilution; monoclonal recombinant rabbit anti-human Beclin-1 antibody (EPR20473, Abcam) at 1:50 dilution; and polyclonal rabbit anti-human LC3B antibody (ab4839zhen, Abcam) at 1:200 dilution. The percentage of positive cells was recorded. The microscope magnification was x400. For immunohistochemical interpretation, we used the following score: negative (0) expression – without positive cells or with a single positive cell (<1%); low (1+) expression – less than 10% positive cells; moderate (2+) expression – 10%-50% positive cells; and strong (3+) expression – more than 50% positive cells (18).

### Statistical analysis

Continuous variables are presented as mean ± standard deviation (SD), while categorical variables are presented as counts and percentages. The χ^2^ test was used to assess the differences in categorical data between study groups. Kendall’s tau b correlation coefficient was calculated to assess the degree of correlation between categorical data. The significance level was set to *P* < 0.05. Statistical analysis was performed with IBM SPSS, version 20.0 (IBM Corp. Armonk, NY, USA).

## Results

### Demographic and histopathological characteristics

The study enrolled 328 women with ovarian tumors (Table 1). The mean age was 52.4 ± 15.8 years (age range: 15-84 years). Women with APT and BOT were significantly younger (*P* < 0.001) than women with EOC. In the EOC group, there were more menopausal women than in the other two groups (*P* < 0.001). The groups significantly differed in the distribution of histological tumor types (*P* < 0.001), with serous tumors being the most common histological type.

**Table 1:** Characteristics of patients with different types of ovarian tumors

	Total n = 328	Epithelial ovarian carcinoma, n = 122	Atypical proliferative tumor, n = 42	Benign ovarian tumor, n = 164
Age, mean ± standard deviation (years)	52.4 ± 15.8	61.8 ± 10.1	45.8 ± 12.2	47.2 ± 15.6
Menopause, n (%)
yes	199 (60.7)	106 (86.9)	20 (47.6)	73 (44.5)
no	129 (39.3)	16 (13.1)	22 (52.4)	91 (55.5)
Histological type, n (%)
serous	206 (62.8)	103 (84.4)	25 (59.5)	78 (47.6)
mucinous	112 (34.2)	10 (8.2)	16 (38.1)	86 (52.4)
endometrioid	10 (3.0)	9 (7.4)	1 (2.4)	0 (0)

### Immunohistochemical analysis of p62, LC3, and Beclin1

**Figure 1:**
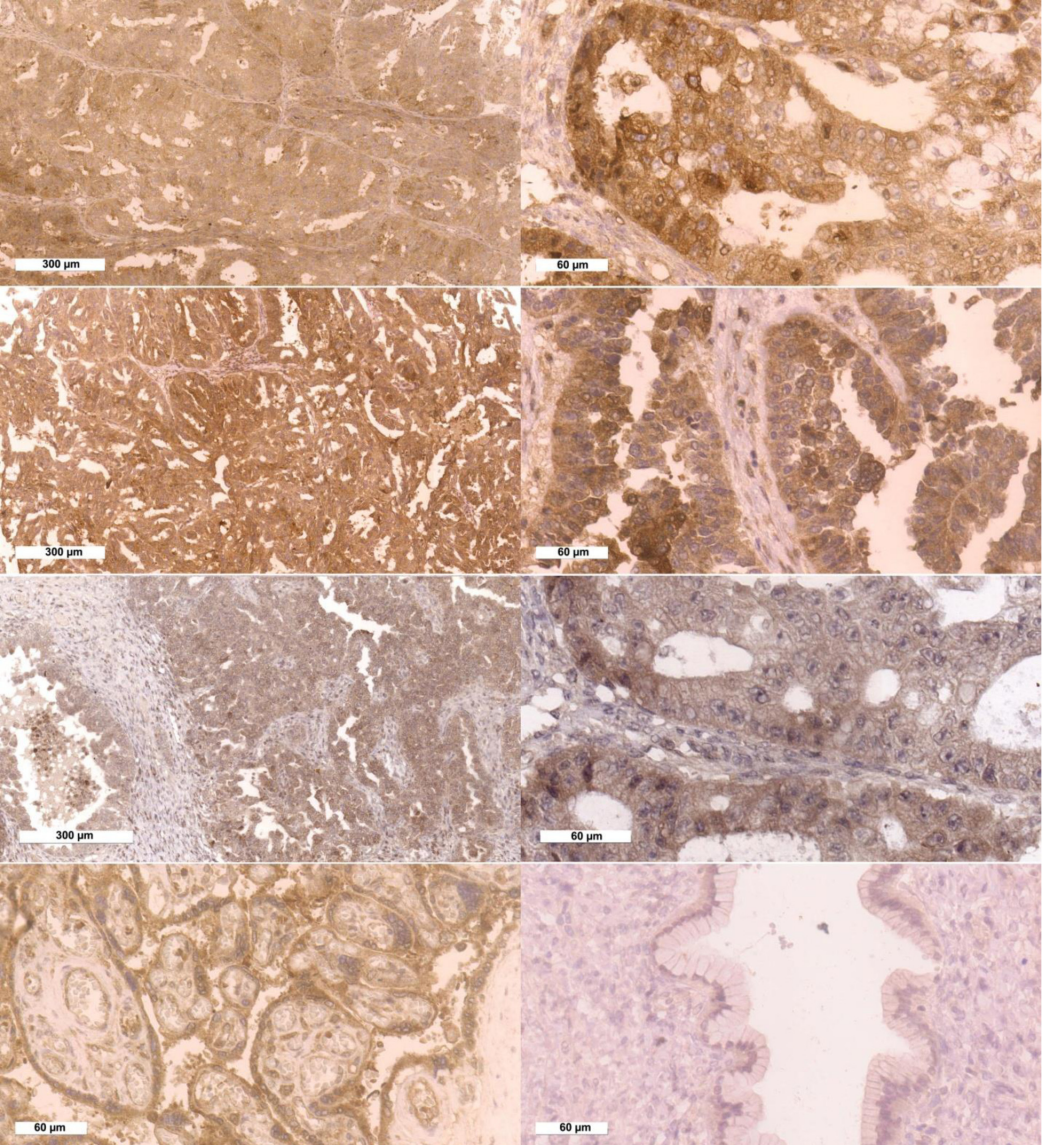
Expression of autophagy markers in tumor cells of epithelial ovarian carcinomas. A. p62 expression (x100); B. p62 expression (x400); C. LC3 expression (x100); D. LC3 expression (x400); E. Beclin1 expression (x100); F. Beclin1 expression (x400); G. Placental tissue as a positive control for immunostaining (x400); H. Benign epithelial ovarian tumor (mucinous) as a negative control (x400).

The expression of p62, LC3, and Beclin1 was analyzed in 328 patients with epithelial ovarian tumors, both in central and invasive tumor segments. The results of immunohistochemical analysis for representative samples with different expression levels of the analyzed markers are shown in Figure 1.

The markers’ expression significantly correlated between central and invasive tumor parts (*P* < 0.001), so only the values for central tumor parts were used in further analyses. There was a strong positive correlation between p62 and LC3 expression, while both markers correlated negatively with Beclin1 expression. The study groups significantly differed in the expression of p62, LC3, and Beclin1 (Table 2). The expression of all three markers was highest in EOC and lowest in BOT.

**Table 2:** Expression of p62, LC3, and Beclin1 in central part of the tumor in patients with ovarian carcinoma

Marker score	No. (%) of patients with			P*
	epithelial ovarian carcinoma	atypical proliferative tumor	benign ovarian tumor	
p62				
0	0 (0)	6 (14.3)	149 (90.9)	all <0.001
1+	3 (2.5)	21 (50.0)	15 (9.1)	
2+	43 (35.2)	15 (35.7)	0 (0)	
3+	76 (62.3)	0 (0)	0 (0)	
LC3				
0	0 (0)	9 (21.4)	149 (90.9)	all <0.001
1+	3 (2.5)	22 (52.4)	15 (9.1)	
2+	35 (28.7)	11 (26.2)	0 (0)	
3+	84 (68.9)	0 (0)	0 (0)	
Beclin1				
0	0 (0)	12 (28.6)	162 (98.8)	all <0.001
1+	15 (12.3)	21 (50.0)	2 (1.2)	
2+	60 (49.2)	9 (21.4)	0 (0)	
3+	47 (38.5)	0 (0)	0 (0)	

*χ^2^ test.

### Association of p62, LC3, and Beclin1 expression with histopathological parameters in EOC

The association of p62, LC3, and Beclin1 expression with histopathological parameters in central tumor parts was assessed in the EOC group (Table 3). For this analysis, patients with HGSC, the most common and the most aggressive EOC form, were compared with all other histological subtypes of EOC (low-grade serous, mucinous, and endometrioid carcinoma). The expression of each marker was significantly associated with tumor histological type, stage, and differentiation (*P* < 0.001). While p62 and LC3 were more prominently expressed in patients with HGSC, Beclin 1 expression was lower in HGSC and more prominent in other histological types. The expression of p62 and LC3 was higher in higher tumor stages (3,4) than in lower tumor stages (1,2). The opposite was observed for Beclin1 expression. Tumor differentiation positively correlated with p62 and LC3 expression, and negatively with Beclin1 expression. p62 and LC3 expression correlated significantly with lymphovascular invasion, necrosis, and lymphocytic infiltrate (peritumoral and intratumoral) (*P* < 0.001).

**Table 3:** The association of p62, LC3, and Beclin1 expression with histopathological parameters in patients with epithelial ovarian carcinoma

	Tumor histological type		Tumor stage		Tumor differentiation		
HGSC*	non-HGSC	1 and 2	3 and 4	1	2	3

p62, n (%)

1+

0 (0)

3 (9.7)

3 (7.7)

0 (0)

3 (20.0)

0 (0)

0 (0)

2+

22 (24.2)

21 (67.7)

36 (92.3)

7 (8.4)

12 80.0)

6 (50.0)

25 (26.3)

3+

69 (75.8)

7 (22.6)

0 (0)

76 (91.6)

0 (0)

6 (50.0)

70 (73.7)

P^†^

all <0.001

all <0.001

all <0.001

LC3, n (%)

1+

0 (0)

3 (9.7)

3 (7.7)

0 (0)

3 (20.0)

0 (0)

0 (0)

2+

17 (18.7)

18 (58.1)

32 (82.1)

3 (3.6)

12 (80.0)

3 (25.0)

20 (21.1)

3+

74 (81.3)

10 (32.2)

4 (10.2)

80 (96.4)

0 (0)

9 (75.0)

75 (78.9)

P^†^

all <0.001

all <0.001

all <0.001

Beclin1, n (%)

1+

12 (13.2)

3 (9.7)

3 (7.7)

12 (14.5)

3 (20.0)

0 (0)

12 (12.6)

2+

49 (53.8)

11 (35.5)

8 (20.5)

52 (62.6)

8 (53.3)

2 (16.7)

50 (52.6)

3+

30 (33.0)

17 (54.8)

28 (71.8)

19 (22.9)

4 (26.7)

10 (83.3)

33 (34.8)

P^†^

all <0.001

all <0.001

all <0.001

*HGSC – high-grade serous carcinoma.

†χ^2^ test.

## Discussion

A combined analysis of p62, LC3, and Beclin1 provided insight into the autophagy status of different ovarian tumors and demonstrated a potential prognostic value of these markers. The major study finding is the association of a specific pattern of p62, LC3, and Beclin1 expression with the degree of tumor malignancy, histological type, stage, and differentiation in EOC.

All markers analyzed in this study have cytoplasmic expression. IHC analysis of LC3 and p62 usually shows “dot-like” cytoplasmic staining in various cancer cells (12). Although some studies describe diffuse cytoplasmic expression of these markers in tumor cells, most researchers support spot cytoplasmic marker expression as the most reliable indicator of the autophagy process (19). p62 expression should be interpreted with caution, since it is influenced by posttranscriptional and posttranslational cell modifications. Regardless of this, p62 expression has been confirmed as a marker of a large number of cancers (13,19,20). Since the LC3 protein is expressed on the autophagosome membrane, its “dot-like” cytoplasmic IHC staining is expected. The expression of the LC3 marker is stable even in the later stages of autophagy when autophagosomes are not observed (21,22). Beclin1 separates from the Bcl-2 molecule during autophagy initiation and fuses with Vps34 molecule, which is also detected as “dot-like” cytoplasmic staining. Alternatively, Beclin1 expression is detected as diffuse cytoplasmic or nuclear staining. Variable expression of autophagy markers warrants the use of combined markers, even if these markers have the same or similar function (5,12,15,19,22).

This study found a strong positive correlation between p62 and LC3 expression, while both markers were negatively correlated with Beclin1, which agrees with previous findings (12,23). The obtained results are expected considering the simultaneous functions of p62 and LC3 at the molecular level (12). To our knowledge, this is the first study to perform IHC staining for each of the analyzed markers in both central and invasive tumor parts. Since the marker expression in the central parts strongly correlated with the expression in the invasive tumor parts, only the value for central tumor parts was used for further analyses.

The expression profile of p62, LC3, and Beclin1 correlated with the degree of tumor malignancy. An association between significant LC3 marker expression and higher tumor aggressiveness was also shown in other cancer types (5,14,19). In our study, the expression of all three markers was highest in EOC, and lowest in BOT, similar to the findings of other studies (24,25). A low p62 expression was previously associated with EOC recurrence, metastasis, and drug resistance (13). Some studies described a high cytoplasmic and low nuclear expression of p62 in EOC with aggressive clinical course and poor prognosis (5,20), which is similar to our results.

The expression of Beclin1 molecules is negatively correlated with the expression of Bcl-2 protein (26). Since Beclin1 protein interacts with antiapoptotic molecules, the loss of its expression is associated with a poor prognosis, as antiapoptotic intracellular signaling pathways in cancer cells are promoted (21,26,27). The heterogeneity of Beclin1 expression in our EOC patients could be explained by an associated function of Beclin1 molecule outside of its involvement in autophagy (28). Higher Beclin1 expression is reported to be associated with a better EOC outcome (29). This finding could be explained by the tumor-suppressing function of the *BECN1* gene, which arrests the cell cycle, inhibits cell proliferation, and promotes autophagy and apoptosis (24,29). In line with this, the loss of Beclin1 expression was reported to be an independent indicator of poor prognosis in EOC (28). While the expression of LC3 as a single marker does not entail sufficient prognostic value in EOC, in combination with Beclin1, it could be of prognostic significance (29).

Our study showed an association of all three analyzed markers with histological type, tumor stage, and tumor differentiation in EOC. Although the expression of Beclin1 was heterogenous, unlike the expression of p62 and LC3, it was significantly correlated with the analyzed histopathology parameters. The expression of p62 and LC3 was more prominent in HGSC compared with other histology types. While p62 and LC3 expression was associated with higher tumor stages and tumor grades, the opposite was found for Beclin1. These findings agree with the results of other studies (15,26,28). Low-grade ovarian carcinomas express Beclin1 more frequently than high-grade ovarian carcinomas (21). Such expression pattern indicates that low levels of autophagy are related to ovarian cancer progression, while upregulation of autophagy is associated with less aggressive histological types of ovarian cancer (21,27). On the other hand, some studies show a positive correlation of Beclin1 expression with tumor aggressiveness (15). Other studies also found a positive correlation between Beclin1 expression and tumor differentiation, suggesting Beclin1 to be a protective factor in EOC (24).

One of the main study limitations is the lack of patient follow-up. Autophagy is closely related to many intracellular mechanisms. Analysis of any autophagy-associated regulation process could improve the strength of this study.

Since autophagy mechanisms are activated by chemotherapeutic drugs, high Beclin1 expression in patients who received chemotherapy indicates the activation of autophagy in tumor cells, which may lead to the development of treatment resistance (10,15). High LC3 expression is related to a poor therapy response to platinum chemotherapy in the treatment of patients with aggressive EOC types (30). Therefore, a combined use of autophagy inhibitors and chemotherapy in EOC should be considered in the treatment of ovarian carcinomas (5,10,15). The use of p62, LC3, and Beclin1 expression as prognostic and predictive markers in EOC could contribute to a more effective therapy and clinical management of this disease.
